# A high-resolution 7-Tesla fMRI dataset from complex natural stimulation with an audio movie

**DOI:** 10.1038/sdata.2014.3

**Published:** 2014-05-27

**Authors:** Michael Hanke, Florian J. Baumgartner, Pierre Ibe, Falko R. Kaule, Stefan Pollmann, Oliver Speck, Wolf Zinke, Jörg Stadler

**Affiliations:** 1 Department of Psychology II, University of Magdeburg, Magdeburg, Germany; 2 Center for Behavioral Brain Sciences, Magdeburg, Germany; 3 INCF Data-sharing taskforce; 4 Visual Processing Laboratory, Ophthalmic Department, University of Magdeburg, Magdeburg, Germany; 5 Leibniz Institute for Neurobiology, Magdeburg, Germany; 6 Department of Biomagnetic Resonance, University of Magdeburg, Magdeburg, Germany; 7 German Center for Neurodegenerative Disease (DZNE), site Magdeburg, Germany

## Abstract

Here we present a high-resolution functional magnetic resonance (fMRI) dataset – 20 participants recorded at high field strength (7 Tesla) during prolonged stimulation with an auditory feature film (“Forrest Gump”). In addition, a comprehensive set of auxiliary data (T1w, T2w, DTI, susceptibility-weighted image, angiography) as well as measurements to assess technical and physiological noise components have been acquired. An initial analysis confirms that these data can be used to study common and idiosyncratic brain response patterns to complex auditory stimulation. Among the potential uses of this dataset are the study of auditory attention and cognition, language and music perception, and social perception. The auxiliary measurements enable a large variety of additional analysis strategies that relate functional response patterns to structural properties of the brain. Alongside the acquired data, we provide source code and detailed information on all employed procedures – from stimulus creation to data analysis. In order to facilitate replicative and derived works, only free and open-source software was utilized.

## Background & Summary

Prolonged complex naturalistic stimulation is arguably more likely to elicit brain responses that are representative of naturally occurring brain states and dynamics than artificial, highly controlled experiments with a limited number of simplified conditions. One particularly interesting aspect of natural stimulation using movies is the temporal synchronicity of changes in the response pattern across individual brains, presumably caused by synchronous temporal dynamics of underlying neuronal processes.

For example, it has been shown that gaze position while watching a movie is highly correlated across human viewers, and that there is even a moderate inter-species correlation between humans and monkeys^1,2^. While the inter-species correlation is probably driven by bottom-up attention due to resembling basic saliency processing for all primates^3^, at least for human perception stimulus aspects beyond basic visual properties are likely to play a role. The claim is consistent with the finding that human observers show a higher covariance of eye movements when watching movie trailers compared to out-of-context natural dynamic scenes^4^. Distortion of the natural sequence by arbitrary cuts and scrambling of the resulting segments causes a systematic drop in correlation across viewers^5^. It seems that semantically richer and more coherent movie stimuli lead to a tighter synchronization of cognitive processes across individual brains. These results from eye-tracking experiments are supported by fMRI studies that show a high correlation of blood oxygenation level dependent (BOLD) time courses in early visual and auditory sensory cortices as well as in higher association cortices between subjects and species during movie watching^6–10^.

Algorithms have been developed that utilize inter-individual synchronicity of changes of the brain state over time to align fMRI data from individual brains into a group space based on functional connectivity patterns^11^ and BOLD time-series correlation^12^. Haxby and colleagues were able to demonstrate that temporally synchronous patterns can be used to transform brain response patterns of individual brains into a high-dimensional representational space with common dimensions for all brains^13^. This technique enables group analyses of distributed activation patterns at the same level of detail and accuracy as the analysis of idiosyncratic patterns of an individual brain. Uniformly, these studies find that deriving inter-individual alignment from fMRI data recorded while participants watch movies yields transformations that are of greater general validity when tested on data from controlled experiments. This is further evidence that movies elicit brain response patterns and dynamics that are representative for naturally occurring neuronal processes.

At this point, the literature only offers an incomplete picture regarding what kind of cognitive processes are associated with the observed inter-individual synchronicity. For example, it remains an open question how much of this synchronicity is dependent on a time-locked *visual* stimulus that is processed, largely in parallel, in a bottom-up fashion and provides relatively comprehensive information about the environment in which movie scenes take place. In order to approach this question, it is necessary to observe dynamics of brain activity during processing of a semantically equally rich stimulus that is likely to engage an equivalently large number of cognitive processes – but without visual stimulation.

We believe that an audio description of a movie matches these criteria. An audio description produced for a visually impaired audience is, in contrast to an audio book, largely identical to the original soundtrack of a movie. Consequently, it maintains the complexity and richness of the “soundscape” of the movie. The soundtrack differs, however, in that an (additional) voice-over narrator verbally describes essential aspects of the visual scenery. Compared to an audio-visual movie, this is a stimulus that leaves a much larger margin for inter-individual differences in imagining scenery, as well as actors’ character and personality traits, while still preserving the time-locked presentation of information to a listener. At the same time, an auditory stimulus limits the effect of an attentional focus on the selection of a subset of simultaneously occurring auditory events, in contrast to the selection of different parts of the visual field.

In order to minimize the difference between the original audio-visual movie and its audio-only description, we chose the drama movie “Forrest Gump” as a stimulus (description: “The story depicts several decades in the life of Forrest Gump, a naïve and slow-witted yet athletically prodigious native of Alabama who witnesses, and in some cases influences, some of the defining events of the latter half of the 20th century in the United States.”; Wikipedia). The storyline of this movie is already carried by an off-screen narrator, hence the additional voice-over audio-description can primarily focus on the description of visual scenery and facial expressions without adding differentiating aspects regarding how the story is told. Moreover, the soundtrack of “Forrest Gump” contains several dozen excerpts of contemporary music from the past few decades, thus further enriching the stimulus with a non-speech dimension. Lastly, the story of “Forrest Gump” makes numerous references to events in recent history that participants are likely to have prior knowledge about and, consequently, may engage similar retrieval processes of long-term memory. In contrast to a related study^14^, this may include retrieval of information encoded more than a decade before the first exposure to this movie.

**Figure 1:**
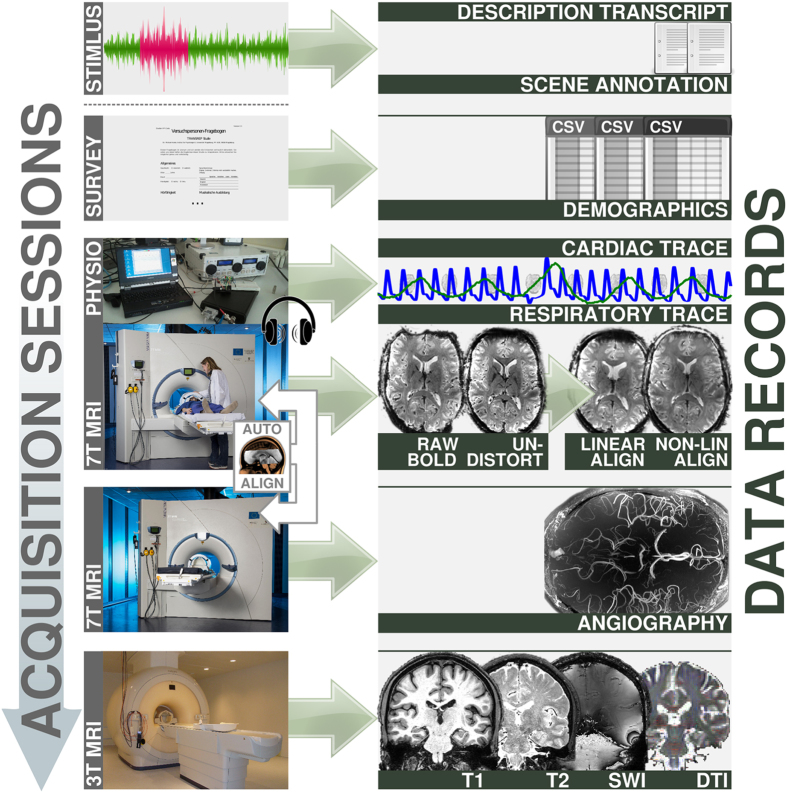
Data acquisition workflow and relation to data records. Acquisition was split into three imaging sessions and a survey. Physiological measurements were recorded simultaneous with the functional MRI during auditory stimulation. MRI data acquistion at 7 Tesla was performed with partial brain coverage. The measurement field-of-view was automatically aligned across sessions and participants. (7 T MRI photo courtesy of the Center for Behavioral Brain Sciences/D. Mahler; 3 T MRI photo courtesy of the Leibniz Institute for Neurobiology/A. Fügner).

We collected fMRI data for the entire duration of a two-hour presentation of this movie. Consequently, this dataset may also be the most comprehensive consecutive sampling of fMRI brain activity patterns for natural language processing. fMRI data were recorded at a high field strength of 7 Tesla and a high spatial resolution of 2.75 mm^3^, accommodating for the relatively small size of the auditory cortices. In order to associate findings from functional data analyses with structural aspects of the brain, a versatile set of auxiliary data has been acquired. This includes high-resolution structural scans (T1- and T2-weighted), localizers for blood vessels and diffusion tensor imaging data. Additionally, physiological measures were recorded simultaneously with fMRI data acquisition to enable effective noise modelling (see Figure 1 for an overview).

An initial analysis indicates that we were able to capture congruent responses across individuals in a large extent of bilateral superior temporal cortex, as well as around Broca’s area. Reflections of these inter-individual similarities are visible in both voxel-wise time-series correlations as well as 2nd-order pattern isomorphisms^15^. Consequently, we believe that these data will be instrumental in shedding light on the nature of the underlying cognitive processes that lead to synchronous brain activity dynamics and can help to extend our knowledge about representational spaces of brain areas beyond the visual processing stream.

Study and protocols were designed to maximize data quality as well as to facilitate replicative and derived works. The large variety of auxiliary measurements allows for a large variety of possible analysis strategies including, but not limited to, a comparison of structural and “functional” connectivity. Given its public status, this dataset can also be used to benchmark and compare algorithms for functional parcellation, physiological noise reduction and tissue segmentation to name but a few.

We hope that our reproducible stimulus and acquisition procedure will be used by independent researchers to extend the scope of this dataset by adding data from participants with different cultural backgrounds, a different spoken language or an audio-visual stimulus. We have already begun to acquire more data and aim to add additional modalities in the future. We invite interested researchers to coordinate with us on creating a high-dimensional brain response based annotation of this movie stimulus.

## Methods

### Participants

Twenty right-handed participants (age 21–38 years, mean age 26.6 years, 12 male) responded to a bulletin posting and volunteered for this study. Native language of all participants was German. They all reported to have normal hearing without permanent or current temporary impairments and no known history of neurological disorders. No formal test of their hearing capabilities was conducted. All but three participants reported to have seen the movie “Forrest Gump” before at least once; the most recent viewing (prior to the experiment) ranged from two weeks to more than a decade. Participants with prior experience had seen the movie on average three times. Two participants had also heard the audio description before.

Participants were fully instructed about the nature of the study, and gave their informed consent for participation in the study as well as for publicly sharing all obtained data in anonymized form. They were paid 100 EUR for their participation. The study was approved by the ethics committee of the Otto-von-Guericke-University of Magdeburg, Germany.

### Procedures

After participants volunteered for the study, each filled out a questionnaire on basic demographic information, musical preference, proficiency and education, as well as familiarity with the “Forrest Gump” movie.

MRI data acquisition was split into three sessions per participant. While functional MRI data were recorded at 7 Tesla, most structural images were obtained using a 3-Tesla scanner on a different day. Due to the length of the movie recording session, acquisition of functional and structural images at 7 Tesla was split into two separate sessions. The order of recording sessions was arbitrary and not constant across participants, although all participants happened to participate in the sessions at 7 Tesla first. Scanning sessions were at least two days apart, but for some participants the recording sessions were spread over several weeks.

At the start of the movie recording session, while auxiliary scans were performed, participants listened to music from the movie’s closing credits segment. During this time, the optimal stimulus volume was determined for each participant individually. Participants were instructed to “maximize the volume of the stimulus without it becoming unpleasantly loud or causing acoustic distortions by overdriving the loudspeaker hardware”. After the start of the movie, participants still had the ability to change the volume, but very few made use of this. Those who did only performed adjustments within the first few seconds of the first movie segment; the volume remained constant across all movie segments otherwise.

All eight movie segments were presented individually in chronological order in two sessions of four segments each. Between sessions, participants left the scanner for a break with a flexible duration. On average, participants chose to continue with the second session after approximately 15 min.

After every movie segment, when the scanner was stopped, participants were asked to rate the acoustic quality of the preceding segment (“How well did you perceive this part?”) on a scale from 1 (very poor) to 4 (very good). Participants responded by making two responses using a 2-button response board with their right hand (first response: “good” vs. “bad”; second response: “rather” vs. “very” to further qualify the previous response). This rating was followed by a brief break, and the recording continued as soon as the participant requested to continue by pressing a button.

Participants were instructed to inhibit any physical movements, as best they could, throughout the recording sessions. At the same time, they were informed to feel free to perform eye-movements, if they were so inclined, and that it was not required to maintain fixation or to keep the eyes open. Other than that, participants were instructed to simply “enjoy the movie”.

For all structural scans, participants were given no task other than to move as little as possible. They were given the option to listen to music of their choice.

### Stimulus

Participants listened to a German audio-description (Koop, Michalski, Beckmann, Meinhardt & Benecke, produced by Bayrischer Rundfunk, 2009) of the movie “Forrest Gump” (R. Zemeckis, Paramount Pictures, 1994) as broadcast as an additional audio track for visually impaired listeners on Swiss public television. The audio content is largely identical to the dubbed German soundtrack of the movie except for interspersed narrations by a male speaker who describes the visual content of a scene. These descriptions take place when there is no dialog, off-screen speech or other relevant audio-content in the movie. During narrations, the volume of the original soundtrack is reduced so that they are clearly audible. To aid reproducibility and further analysis, the audio description soundtrack was temporally aligned to the audio track of the “Forrest Gump” DVD release (Paramount Home Entertainment, Germany, 2002; PAL video, DE103519SV) by shifting the audio description to match waveform envelope shapes in audacity^16^. For this purpose, the audio track of the DVD was extracted using the Handbrake software^17^ and, together with the audio description, both were converted into the “Free Lossless Audio Codec”^18^ while down-mixing multi-channel audio to stereo.

During echo planar imaging (EPI), the MRI scanner emits a significant amount of noise, with major frequency components being: EPI sequence base frequency 641 Hz (due to an echo spacing of 0.78 ms) and its harmonics at 1923 Hz and 3205 Hz, as well as gradient coil resonance frequencies at 540 Hz and 1000 Hz. In order to achieve optimal saliency of the audio stimulus under these conditions, the audio signal was processed by a series of filters. First, a multi-band equalizer was used to implement a high-pass filter (−40 db attenuation in the 50 Hz band and −5 db at 100 Hz) to remove low frequencies that would have caused acoustic distortions in the headphones at high volume. This filter was followed by a 5 db volume gain. Finally, a Dyson compressor (25 db limit, 250 ms release time, 0.4 fast compression ratio, 0.6 compression ratio) and a lookahead limiter (17 db input gain, −3 db limit, 500 ms release time) were used to reduce the dynamic range of the audio signal and, consequently, relatively silent parts of the movie (*e.g.*, sounds of crickets at night) became approximately as loud as scenes with originally high volume (*e.g.*, explosions).

**Figure 2:**
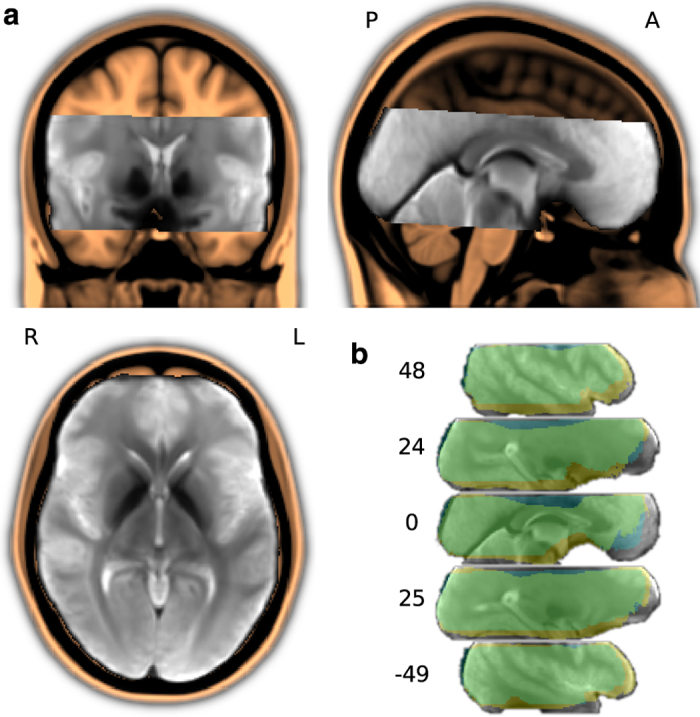
Field-of-view for the fMRI data acquisition. (**a**) Custom T2* EPI group template (in gray), linearly aligned to and overlaid on the MNI152 T1-weighted head template (in amber). The EPI template was created by an iterative procedure with four linear and ten non-linear alignment steps out of one sample volume per run and brain (a total of 152 images; images from participant 10 were excluded; see Table 3, slice cut point: anterior commissure at MNI 0,0,0 mm). (**b**) Intersection masks after linear and non-linear anatomical alignment of all mean volumes for all individual fMRI runs across all participants. The linear intersection mask is depicted in blue, non-linear in yellow (overlap in green). Coordinates are in MNI millimeters.

In order to keep the fMRI recording session under two hours, the movie was slightly shortened by removing a few scenes that were less relevant to the major plot. Table 1 lists all parts of the movie that comprise the actual stimulus. The shortened movie was further split into eight, approximately 15 min long, segments. Except for the first movie segment, each segment started with a movie snippet of at least six seconds immediately preceding the movie scene boundary used to split the segments. The start of each segment was synchronized with the MRI scanner’s volume acquisition trigger signal. All segments, except for the first one, started by repeating the equivalent of three fMRI volumes from the previous segment. Volume acquisition was time-locked across segments with respect to the movie time. The exact transition point was determined by taking a scene boundary and locating the closest multiple of the volume acquisition time (2 s) prior to this boundary. A total of five volumes after this transition point were recorded at the end of each segment. The stimulus intensity was faded in (1 s) and out (4 s) at the start and end of each segment. Time codes for scene boundaries that were used to determine the segment transition points were: 00:14:52.19, 00:32:00.13, 00:46:47.0, 01:04:58.11, 01:22:18.11, 01:39:36.1, 01:58:44.8, 02:09:52.2 (timestamp format is identical to the one in Table 1; all time codes refer to the original movie and not the shortened version). Movie closing credits (everything after the last boundary time code) were not shown. Details on movie segment presentation and transitions are shown in Figure 2a.

### Movie segment creation

All video/audio editing was performed using the “melt” command line video editor^19^ on a PC with a Debian operating system. This section lists the exact commands used to produce the stimulus for each of the eight movie segments. This includes producing the stimulus cut from the DVD version, as well as all filtering done to the audio signal. All processing was implemented using the MLT framework^19^ while utilizing several LADSPA audio processing plugins (Steve Harris, Ushodaya Enterprises Limited). For the sake of a more compact presentation common options for audio filtering and output format specifications have been replaced by placeholders. Audio filtering options (AUDIOFILTER) were:


# Multiband EQ
-attach-track ladspa.1197 0=− 40 1=−5
# Volume gain
-attach-track volume gain=5dB
# Dyson compressor
-attach-track ladspa.1403 0=−25 1=0.25 2=0.4 3=0.6
# Fast lookahead limiter
-attach-track ladspa.1913 0=17 1=−3 2=0.5

Video output specification options (OUTPUTSPEC) were:


f=matroska s=940×400 aspect=2.35 vcodec=libx264 b=500 k acodec=libmp3lame ab=256 k

Individual movie segments were rendered by executing the following commands:

Segment 1 (length: 902 s)


melt -video-track \
gray.png out=25 fixation.png out=14000 -mix 25 -mixer luma \ 
fixation.png out=8550 gray.png out=100 -mix 250 -mixer luma \ 
-audio-track AUDIOFILTER \ 
silence_1s.flac ad_ger_stereo.flac in=0 out=22550 -mix 25 -mixer mix:-1 \ 
silence_5s.flac -mix 100 -mixer mix:-1 \ -consumer avformat:fg_ad_seg0.mkv OUTPUTSPEC

Segment 2 (length: 882 s)


melt -video-track \ 
gray.png out=25 fixation.png out=10162 -mix 25 -mixer luma \ 
fixation.png out=11888 gray.png out=100 -mix 250 -mixer luma -audio-track AUDIOFILTER \ 
silence_1s.flac ad_ger_stereo.flac in=22150 out=32312 -mix 25 -mixer mix:-1 \ 
ad_ger_stereo.flac in=36349 out=48237 silence_5s.flac -mix 100 -mixer mix:-1 \ 
-consumer avformat:fg_ad_seg1.mkv OUTPUTSPEC

Segment 3 (length: 876 s)


melt -video-track \ 
gray.png out=25 fixation.png out=9961 -mix 25 -mixer luma \ 
fixation.png out=11939 gray.png out=100 -mix 250 -mixer luma \ 
-audio-track AUDIOFILTER \ 
silence_1s.flac ad_ger_stereo.flac in=47837 out=57798 -mix 25 -mixer mix:-1 \ 
ad_ger_stereo.flac in=58470 out=70409 silence_5s.flac -mix 100 -mixer mix:-1 \ 
-consumer avformat:fg_ad_seg2.mkv OUTPUTSPEC

Segment 4 (length: 976 s) 


melt -video-track \ 
gray.png out=25 fixation.png out=14000 -mix 25 -mixer luma \ 
fixation.png out=1988 fixation.png out=8412 gray.png out=100 -mix 250 -mixer luma \ 
-audio-track AUDIOFILTER \ 
silence_1s.flac ad_ger_stereo.flac in=70009 out=85997 
-mix 25 -mixer mix:-1 \ 
ad_ger_stereo.flac in=89293 out=97705 silence_5s.flac -mix 100 -mixer mix:-1 \ 
-consumer avformat:fg_ad_seg3.mkv OUTPUTSPEC

Segment 5 (length: 924 s) 


melt -video-track \ 
gray.png out=25 fixation.png out=14000 -mix 25 -mixer luma \ 
fixation.png out=6046 fixation.png out=3054 gray.png out=100 -mix 250 -mixer luma \ 
-audio-track AUDIOFILTER \ 
silence_1s.flac ad_ger_stereo.flac in=97305 out=117351 -mix 25 -mixer mix:-1 \ 
ad_ger_stereo.flac in=120616 out=123670 silence_5s.flac -mix 100 -mixer mix:-1 \ 
-consumer avformat:fg_ad_seg4.mkv OUTPUTSPEC

Segment 6 (length: 878 s) 


melt -video-track \ 
gray.png out=25 fixation.png out=14000 -mix 25 -mixer luma \ 
fixation.png out=4187 fixation.png out=3763 gray.png out=100 -mix 250 -mixer luma \ 
-audio-track AUDIOFILTER \ 
silence_1s.flac ad_ger_stereo.flac in=123270 out=141457 -mix 25 -mixer mix:-1 \ 
ad_ger_stereo.flac in=145869 out=149632 silence_5s.flac -mix 100 -mixer mix:-1 \ 
-consumer avformat:fg_ad_seg5.mkv OUTPUTSPEC

Segment 7 (length: 1084 s) 


melt -video-track \ 
gray.png out=25 fixation.png out=3028 -mix 25 -mixer luma \ 
fixation.png out=14000 fixation.png out=10072 gray.png out=100 -mix 250 -mixer luma \ 
-audio-track AUDIOFILTER \ 
silence_1s.flac ad_ger_stereo.flac in=149232 out=152260 -mix 25 -mixer mix:-1 \ 
ad_ger_stereo.flac in=154244 out=178316 silence_5s.flac -mix 100 -mixer mix:-1 \ 
-consumer avformat:fg_ad_seg6.mkv OUTPUTSPEC

Segment 8 (length: 675.04 s) 


melt -video-track \ 
gray.png out=25 fixation.png out=14000 -mix 25 -mixer luma \ 
fixation.png out=2876 gray.png out=100 -mix 250 -mixer luma \ 
-audio-track AUDIOFILTER \ 
silence_1s.flac ad_ger_stereo.flac in=177916 out=194792 -mix 25 -mixer mix:-1 \ 
silence_5s.flac -mix 100 -mixer mix:-1 \ 
-consumer avformat:fg_ad_seg7.mkv OUTPUTSPEC

### Stimulation setup

Participants listened to the movie stimulus using custom-built in-ear headphones. In order to maximize participants’ comfort in the narrow head coil and maintain a high audio quality, the headphones use a combination of electrostatic loudspeakers (HP-M01, MR confon GmbH, Magdeburg, Germany) located just outside the head coil, a short air-conducting part consisting of a funnel-like adapter, and an approximately 15 cm long tube that connected a speaker with a communication earplug (Peltor HearPlug, PELTIP1-1, ≈25 db attenuation). This setup significantly reduces the pressure on the auricles, thus avoiding the pain often experienced during imaging sessions, and consequently helped participants maintain a steady head position. Headphones were driven by an MR confon mkII+ fed from an Aureon 7.1 USB (Terratec) sound card through an optical connection.

Visual instructions were presented with an LCD projector (DLA-G150CL, JVC Ltd.) on a rear-projection screen positioned behind the head coil within the magnetic bore. Participants viewed the screen through a mirror attached to the head coil. During the functional scans, the projector presented a medium gray screen with the primary purpose to illuminate a participant’s visual field in order to prevent premature fatigue. The screen contained a solid black “fixation” dot that faded in and out at the beginning and end of a movie segment (see Figure 2a).

Stimulus presentation and response logging were implemented using PsychoPy^20^. The source code of the complete implementation of the experiment is available as Supplementary Material in the data release. PsychoPy was running on a computer with the (Neuro)Debian operating system^21^.

### Response-/synchronization setup

The optical trigger signal emitted by the MRI scanner is converted to a TTL signal. The TTL signal was fed to the physiological recording setup and simultaneously to a Teensy3 microcontroller (PJRC.COM, LLC., Sherwood, OR, USA). The open collector signal from the response board (ResponseBox 1.0, Covilex, Magdeburg, Germany) was also fed into the Teensy3. A simple “teensydurino sketch” was used to convert the signals to USB keyboard events (“t”, “1”, “2”).

### Functional MRI data acquisition

**Figure 3:**
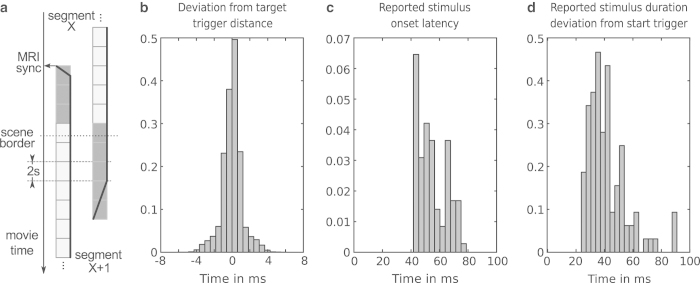
Stimulus synchronization and timing accuracy. (**a**) Schema for movie segment transitions and synchronization with the MRI scanner's volume acquisition trigger signal. The solid black line shows the relative stimulus volume at the beginning and end of a movie segment. (**b**) Histogram of inter-trigger pulse duration deviations from the target duration of 2 s – as recorded by the stimulus software. This is an estimate of the temporal uncertainty of the timing information. Trigger pulses are sent by the MRI scanner at precise intervals of 2 s at the start of each volume. (**c**) Histogram of movie onset latencies (deviation from the respective trigger pulse) as reported by the stimulus software. This is a worst-case estimate that includes all file access latencies and indicates the complete latency until the underlying movie presentation engine reports the start of a movie segment back to the stimulus software. (**d**) Histogram of deviations of movie segment duration from target duration. All histograms are normalized and aggregate information across all participants.

T2*-weighted echo-planar images (gradient-echo, 2 s repetition time (TR), 22 ms echo time, 0.78 ms echo spacing, 1488 Hz/Px bandwidth, generalized autocalibrating partially parallel acquisition (GRAPPA) acceleration factor 3, 24 Hz/Px bandwidth in phase encoding direction) were acquired during stimulation using a whole-body 7-Tesla Siemens MAGNETOM magnetic resonance scanner equipped with a local circularly polarized head transmit and a 32 channel brain receive coil (Nova Medical, Inc., Wilmington, MA, USA). 36 axial slices (thickness 1.4 mm, 1.4×1.4 mm in-plane resolution, 224 mm field-of-view (FoV), anterior-to-posterior phase encoding direction) with a 10% inter-slice gap were recorded in ascending order. This configuration represents a good compromise between spatial resolution, volume coverage and volume acquisition time (Figure 3).

Slices were oriented to include the ventral portions of frontal and occipital cortex while minimizing intersection with the eyeballs. The field-of-view was centred on the approximate location of Heschl’s gyrus. Slice orientation was manually configured for the first pilot recording session only. Subsequent sessions for all participants were automatically aligned to this setup by the scanner software (auto align) using skull and brain landmarks identified from a short 3D scout scan (1.6 mm isotropic resolution, 14 s duration).

A total of 3599 volumes were recorded for each participant (451, 441, 438, 488, 462, 439, 542 and 338 volumes for movie segment 1–8 respectively). EPI images were online-corrected for motion and geometric distortions^22–24^. Auxiliary scans for slice alignment and motion- and distortion-correction were performed at the beginning of the first fMRI recording session and also after the break at the start of the recording for the second half of the movie.

### Physiological recordings

Physiological data were recorded simultaneously with the fMRI data acquisition using a custom setup^25^ and in-house recording software (written in Python). A Nonin 8600 FO pulse oxymeter (Nonin Medical, Inc, Plymouth, MN, USA) was used to measure cardiac trace and oxygen saturation, and a Siemens respiratory belt connected to a pressure sensor (Honeywell 40PC001B1A) captured the respiratory trace. Analog signals were digitized (12 bit analog digital converter, National Instruments USB-6008) at a sampling rate of 200 Hz. The digitizer also logged the volume acquisition trigger pulse emitted by the MRI scanner.

### Structural MRI data acquisition

Five types of structural images were acquired for each participant. Except for the time-of-flight angiography (which was recorded using the same scanner as the functional MRI at 7 Tesla), all images were recorded with a 3-Tesla Philips Achieva equipped with a 32 channel head coil using standard clinical acquisition protocols.

#### T1-weighted image

An image with 274 sagittal slices (FoV 191.8×256×256 mm) and an acquisition voxel size of 0.7 mm with a 384×384 in-plane reconstruction matrix (0.67 mm isotropic resolution) was recorded using a 3D turbo field echo (TFE) sequence (TR 2500 ms, inversion time (TI) 900 ms, flip angle 8 degrees, echo time (TE) 5.7 ms, bandwidth 144.4 Hz/px, Sense reduction AP 1.2, RL 2.0, scan duration 12:49 min).

#### T2-weighted image

A 3D turbo spin-echo (TSE) sequence (TR 2500 ms, TE_*eff*_ 230 ms, strong SPIR fat suppression, TSE factor 105, bandwidth 744.8 Hz/px, Sense reduction AP 2.0, RL 2.0, scan duration 7:40 min) was used to acquire an image whose geometric properties otherwise match the T1-weighted image.

#### Susceptibility-weighted image

An image with 500 axial slices (thickness 0.35 mm, FoV 181×202×175 mm) and an in-plane acquisition voxel size of 0.7 mm reconstructed at 0.43 mm (512×512 matrix) was recorded using a 3D Presto fast field echo (FFE) sequence (TR 19 ms, TE shifted 26 ms, flip angle 10 degrees, bandwidth 217.2 Hz/px, NSA 2, Sense reduction AP 2.5, FH 2.0, scan duration 7:13 min).

#### DTI and field map

Diffusion data were recorded with a diffusion-weighted single-shot spin-echo EPI sequence (TR 9545 ms, TE 80 ms, strong SPIR fat suppression, bandwidth 2058.4 Hz/px, Sense reduction AP 2.0) using 32 diffusion-sensitizing gradient directions with $b=800\frac{s}{mm^{2}}$ (two samples for each direction), 70 slices (thickness of 2 mm and an in-plane acquisition voxel size of 2×2 mm, reconstructed at 1.7×1.7 mm, 144×144 in-plane matrix, FoV 224×248×140 mm). Acquisition duration was 12:38 min.

Immediately following the DTI acquisition, a field map was recorded to aid distortion correction (50 slices, tilted 30 degrees from the AC-PC line, slice thickness/gap=3 mm/0 mm, TR=688 ms, TE1/TE2=4.92/7.38 ms, FOV=240×240, and a 80×80 matrix with resolution of 3×3 mm).

#### Time-of-flight angiography

A 3D multi-slab time-of-flight angiography was recorded at 7 Tesla for the FoV of the fMRI recording. Four slabs with 52 slices (thickness 0.3 mm) each were recorded (192×144 mm FoV, in-plane resolution 0.3×0.3 mm, GRAPPA acceleration factor 2, phase encoding direction right-to-left, 15.4% slice oversampling, 24 ms TR, 3.84 ms TE).

## Data Records

All data records listed in this section are available from the OpenfMRI portal (dataset accession number: ds000113) at http://openfmri.org/dataset/ds000113. A README file with a detailed description of the content of all downloads is also available at this URL. Additional material and information are provided at http://www.studyforrest.org.

Unless noted otherwise, all MRI data files were converted from DICOM to NIfTI format using the mcverter tool that is part of the MRIConvert package^26^. Converter status output was captured in files that carry a _dicominfo.txt suffix in the filename.

In order to de-identify data, information on centre-specific study and subject codes have been removed using an automated procedure. All human participants were given sequential integer IDs. Furthermore all structural images were “de-faced” by applying a mask image that zeroed out all voxels in the vicinity of the facial surface, teeth and auricles. For each image modality this mask was aligned and re-sliced separately. The resulting tailored mask images are provided as part of the data release to indicate which parts of the image were modified by the de-facing procedure (de-face masks carry a _defacemask suffix to the base file name).

If available, complete device-specific parameter protocols are provided for each acquisition modality.

### Participants’ responses

**Location**
demographics.csv

**File format** plain text

Participants’ responses to the questionnaire on basic demographic information, musical preference, proficiency, education and familiarity with the “Forrest Gump” movie are available in a comma-separated value (CSV) file. Data are structured as one line per participant with questionnaire items as columns. A description of all items is given in Table 2. The same file also contains the participants’ responses to the audio quality rating after each movie segment presentation.

### Functional MRI

**Location**
sub<ID>/BOLD/task001_run00[1–8]/bold[[_dico]_dico7Tad2grpbold7Tad[_nl]].nii.gz

**File format** NIfTI, gzip-compressed

**Sequence protocol**
acquisition_protocols/task001_fmri_session[1|2].pdf

fMRI data are available in four different flavours, each stored in an individual 4D image for each movie segment separately. Raw BOLD data are stored in bold.nii.gz. While raw BOLD data are suitable for further analysis, they suffer from severe geometric distortions. BOLD data that have been distortion-corrected at the scanner computer are provided in bold_dico.nii.gz. Two additional flavours of the distortion-corrected data are provided that have been anatomically aligned to each other to facilitate group analyses. Details of the alignment procedure are given in the section on “Technical Validation”. Data aligned by applying an affine transformation only are available in bold_dico_dico7Tad2grpbold7Tad.nii.gz. Data aligned by non-linear warping are available in bold_dico_dico7Tad2grpbold7Tad_nl.nii.gz.

### Motion estimates

**Location**
sub<ID>/BOLD/task001_run00[1-8]/bold_dico_moco.txt

**File format** plain text

Data motion correction was performed within scanner online reconstruction as part of the distortion correction procedure, and the associated motion estimates are provided in a whitespace-delimited 6-column text file (translation X, Y, Z in mm, rotation around X, Y, Z in deg) with one row per fMRI volume for each movie segment separately.

### Physiological recordings

**Location**
sub<ID>/physio/task001_run00[1-8]/physio.txt.gz

**File format** plain text, gzip-compressed

Physiological data were down-sampled to 100 Hz and truncated to start with the first MRI trigger pulse and to end one volume acquisition duration after the last trigger pulse. Data are provided in a four-column (MRI trigger, respiratory trace, cardiac trace and oxygen saturation), space-delimited text file for each movie segment. A log file of the automated conversion procedure is provided in the same directory (conversion.log).

### T1-weighted image

**Location**
sub<ID>/anatomy/highres001.nii.gz

**File format** NIfTI, gzip-compressed

**Sequence protocol**
acquisition_protocols/04-sT1W_3D_TFE_TR2300_TI900_0_7iso_FS.txt

A 3D volumetric image.

### T2-weighted image

**Location**
sub<ID>/anatomy/other/t2w001.nii.gz

**File format** NIfTI, gzip-compressed

**Sequence protocol**
acquisition_protocols/05-sT2W_3D_TSE_32chSHC_0_7iso.txt

A 3D volumetric image.

### Susceptibility-weighted image

**Location**
sub<ID>/anatomy/other/swi001_[mag|pha].nii.gz

**File format** NIfTI, gzip-compressed

**Sequence protocol**
acquisition_protocols/06-VEN_BOLD_HR_32chSHC.txt

Magnitude and phase images are provided as separate 3D volumetric images. Phase images were converted into $\frac{rad}{s}$.

### Diffusion tensor imaging data

**Location**
sub<ID>/dti/dti001.[nii.gz|bvecs|bvals]

**File format** NIfTI, gzip-compressed or plain text

**Sequence protocol**
acquisition_protocols/07-DTI_high_2iso.txt

In contrast to all other imaging modalities, DTI data were converted using dcm2nii (part of MRICron^27^) which also performed extraction of corresponding b-values and b-vectors for diffusion directions. DTI data are provided in a 4D volumetric image, whereas b-values and v-vectors are stored in space-delimited text files.

### Field map

**Location**
sub<ID>/fieldmap/fieldmap001_[mag|pha].nii.gz

**File format** NIfTI, gzip-compressed

**Sequence protocol**
acquisition_protocols/08-field_map.txt

Magnitude and phase images are provided as separate 3D volumetric images. Phase images for all acquired field maps were converted into $\frac{rad}{s}$.

### Time-of-flight angiography

**Location**
sub<ID>/angio/angio001.nii.gz

**File format** NIfTI, gzip-compressed

**Sequence protocol**
acquisition_protocols/angio_session.pdf

A 3D volumetric image.

### Audio description transcript

**Location**
stimulus/task001/annotations/german_audio_description.csv

**File format** plain text

The text and timing of the German audio description cannot be inferred from the Forrest Gump DVD release, therefore we provide a transcript in a comma-separated value table. Each line of the transcript table represents one temporally continuous audio description segment. The first and second columns list start time and end time of a segment. Start and end times were estimated with a target precision of a tenth of a second by manually locating speech onsets and offsets based on the waveform shapes in Audacity. Values in the third column indicate how fast a description was spoken (normal: 1; fast: 2; very fast: 3). The fourth column contains the actual transcript of the spoken description. Special comments wrapped in brackets within the transcript contain hints whether the spoken description interferes with other dialog or indicate the exact temporal location of description segments with respect to effects in the movie soundtrack.

### Movie scene annotations

**Location**
stimulus/task001/annotations/scenes.csv

**File format** plain text

The movie stimulus is structured into 198 scenes. A scene boundary is defined here as a change of location of the depicted setting in the movie, in contrast to a shot boundary that represents an abrupt change of camera field-of-view ( *i.e.*, a “cut”). Based on the visual movie frames, we determined scene boundaries by manually locating them using a video player. The timestamp associated with a boundary (1 s target precision) is stored in the first column of a CSV table. The second column contains a label for the location where a scene is taking place. In the third and fourth column, we also noted whether a scene took place indoors or outdoors (int vs. ext) as well as the time of day (day vs. night).

## Technical Validation

All image analyses presented in this section were performed on the released data in order to test for negative effects of the de-identification procedure on subsequent analysis steps.

During data acquisition, (technical) problems were noted in a log. All known anomalies and their impact on the dataset are detailed in Table 3.

### Stimulus quality assessment

On average, participants rated the acoustic quality of all movie segments as “rather good” (four-point scale: 1: very poor; 2: rather poor; 3: rather good; 4: very good) or better (mean=3.36, std=0.1 across all movie segments). Segment 1 and 6 were rated best (mean=3.47) and segment 2 received the worst rating (mean=3.16). Participants reported that the male narrator of the audio description and the Forrest Gump voice were most salient in the noisy scanner environment. Several participants mentioned increased difficulty of following female voices and voices of children. Some participants reported to have been unable to understand “background-speech” and other aspects related to the acoustic atmosphere of a scene.

### Timing accuracy

In order to assess timing accuracy of the stimulation and synchronicity of physiological data with respect to the fMRI data acquisition, the MR-scanner’s trigger pulses (emitted at the start of each volume acquisition at regular intervals of 2 s) were used as a reference and recorded on the stimulation computer and the system for physiological data acquisition.

#### Stimulus onset latency

The movie segment onset latency relative to the start trigger pulse, as reported by the stimulation software, was always less than 80 ms across all movie segments and participants (Figure 2c). The upper precision bound of this estimate can be determined by the temporal jitter of MR trigger pulses as recorded on the stimulus computer. Across all recording sessions, this jitter was distributed around a zero mean with a range of approximately ±5 ms (Figure 2b).

#### Physiological recordings

The cumulative temporal drift of physiological recordings relative to the fMRI data, as determined by the deviation of inter-trigger pulse distance from the reference of 2 s, was found to be always equal to or less than 30 ms (equivalent to 3 samples at 100 Hz) for any movie segment (≈15 min duration).

### Structural MRI

In order to assess the quality of the T1-weighted and T2-weighted images, Freesurfer version 5.3.0^28^ was used to perform a complete image segmentation and cortical surface reconstruction pipeline (recon-all with default parameters). T2-weighted images were used in this pipeline to improve the estimate of the pial surface boundary. For all participants, Freesurfer was able to perform cortical surface reconstruction fully automatically. Visual inspection aided by Freesurfer’s QATool (version 1.1, available from the Freesurfer Wiki) revealed no obvious reconstruction errors. It should be noted that, in contrast to the recommendations by the Freesurfer authors, T1- and T2-weighted images were not acquired using bandwidth-matched protocols, but instead recording parameters were optimized individually for each modality. This was done to improve the usability of the T2-weighted images for analyses other than surface reconstruction.

### Functional MRI

#### Temporal signal-to-noise ratio

An additional fMRI dataset was acquired in order to aid the assessment of technical noise of the recording setup for this particular choice of parameters in combination with the stimulation procedures. The procedure was identical to the acquisition protocol for human participants. Visual and auditory stimulation were active, the headphones were positioned in the identical location, and the audio volume was set to the mean amplitude observed across all human participants. fMRI data for the complete movie were acquired using a FBIRN gel phantom (http://www.birncommunity.org/tools-catalog/function-birn-stability-phantom-qa-procedures/). The inter-session break between movie segments four and five was of the same approximate length as for human participants.

**Figure 4:**
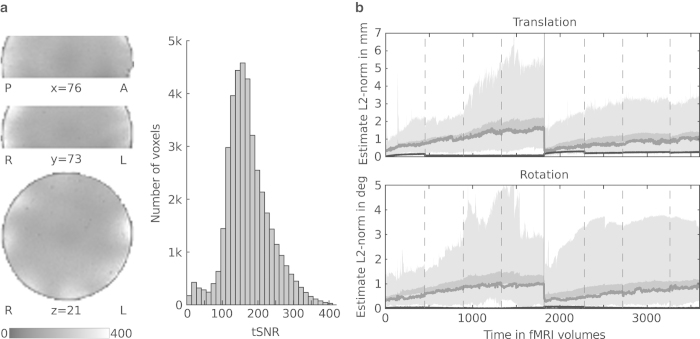
Temporal noise estimates. (**a**) Distribution of the temporal signal-to-noise ratio (tSNR) computed from the temporally de-trended gel phantom dataset. The left side of the panel shows the spatial distribution in arbitrarily selected slices. The right side shows a tSNR histogram across all voxels inside the phantom. (**b**) Motion estimates relative to reference at the beginning of each scan session. The area shaded in light gray depicts the range across participants, while the dark gray area shows the standard error of the mean. The dark gray line indicates the median estimate. Dashed vertical lines indicate run boundaries where participants had a brief break. The solid vertical line indicates the scan session boundary where participants left the scanner for a longer break and a new reference image was recorded afterwards. Data from participant 10 have been excluded as no valid reference image was available. As a reference, the black line shows the estimated motion of the phantom recording.

The temporal signal to noise ratio (tSNR) of the fMRI data, as estimated from the phantom dataset, is within the expected range for this particular parallel imaging acceleration factor and acquisition setup^29^. Figure 4a illustrates the tSNR distribution across the acquisition volume.

#### Participant motion assessment

All but two participants showed very little head motion over the entire length of the recording sessions. The median L2-norm of the estimated translation vector did not exceed 1.8 mm and estimated median L2-normed rotation did not exceed 1 degree. The maximum amplitude of both translation and rotation were lower in the second recording session than in the first half of the movie. Figure 4b depicts the variation of head motion over time, and Table 3 lists movie segments affected by significant motion.

#### EPI group template

In order to facilitate analysis of inter-individual activation patterns, we used an iterative procedure to derive a group-specific template volume for EPI images to aid anatomical alignment across brains (see Figure 3). For 19 participants (data from participant 10 were excluded due to inappropriate distortion correction), we extracted the first volume as a reference image from each movie segment recording, yielding 152 images. All images from an individual brain were aligned to its respective reference image from the first movie segment using a rigid body transformation implemented in MCFLIRT^30^ and averaged to create a template image for each brain. Subsequently, all brains were aligned, by means of an affine transformation using FLIRT^30^, to the one individual brain with the least root mean square difference to the average image across all brains prior to alignment. The initial alignment target volume was slightly upsampled to 1.2 mm isotropic voxel size to account for spatial oversampling across individuals and movie segments. The alignment target for all subsequent iterations was produced by computing the average image across all aligned brains for each respective iteration. Three more iterations with affine transformations were then followed by ten non-linear alignment iterations using FNIRT^31^ global non-linear intensity model with bias and 1 cm warp-field resolution while holding the base affine transformation constant. Finally, the resulting average image was cropped to the region with maximum overlap across individual brains to create the group EPI template volume.

For all subsequent analyses, all data from the 152 movie segments were aligned to this template independently. For each run, the average volume across all time points was computed and aligned to the template through an affine transformation determined by FLIRT while reslicing to 1.2 mm isotropic resolution (“linear alignment”). In addition, this affine transformation was combined with a non-linear warping derived by FNIRT (“non-linear alignment”) and, again, images were resliced to 1.2 mm isotropic resolution. The entire procedure was implemented in the Nipype framework^32^ and the source code of the complete process of generating the group templates is available as Supplementary Material in the data release.

#### Inter-individual response pattern similarity

The primary purpose of this dataset is to study properties of distributed brain response patterns that are common across brains. It is common practice to employ a BOLD time-series similarity measure like correlation to quantify inter-individual response similarities for natural stimulation with a movie (see ref. 6 for an example of this approach).

However, as it becomes increasingly difficult to identify functionally corresponding voxels across brains at high spatial resolution, we did not only assess pattern similarity using voxel-wise time-series correlations of anatomically aligned data. Instead, we employed representational similarity analysis (RSA)^33^ to identify 2nd-order isomorphisms in the response patterns across brains. The premise of RSA is that any two segments of the audio movie which elicit similar neural signal patterns in one brain also elicit similar patterns in another brain; whereas two segments, which lead to distinct patterns in one brain, also evoke dissimilar patterns in another brain.

To do so, we utilized PyMVPA 2.2.0^34,35^ and SciPy 0.7.2^36^ under Python 2.6.6 for data preparation and analysis. Pattern dissimilarity, in this case, was defined as the Pearson correlation distance of all combinations of spatio-temporal response patterns for short non-overlapping segments of the audio movie (a total of 590 12 s segments). After movie-segment-wise pre-processing of the fMRI data (band-pass filter which passes temporal frequency changes with periods between 9 s and 150 s; voxel-wise Z-scoring), response patterns were determined by concatenating all fMRI runs for all movie segments into a single time-series image while removing the last and first four volumes at each boundary connecting the runs. In the resulting time-series, any block of six consecutive non-overlapping volumes represents the spatio-temporal pattern for a movie segment.

Dissimilarity matrices (DSMs) were computed individually for each brain using a searchlight mapping approach^37^ where a small spherical neighbourhood (radius 4 voxels, 4.8 mm, max. 257 voxels) was centred on voxels in the intersection mask of the field-of-view across all brains after linear and non-linear alignment to the group template image. To account for the redundancy of overlapping searchlight spheres, the centre voxels were selected according to a hexagonal close packing structure with a sphere radius of 2 voxels (approx. 2% of all voxels were selected as seed voxels).

The inter-brain pattern consistency index for each searchlight location after alignment was defined as the Spearman rank correlation between the vectorized DSMs for any pair of brains (degrees of freedom=173753). Participants 4 and 10 were excluded from this analysis due to missing data (see Table 3), hence a total of 153 pairwise correlations were computed for the 18 remaining brains at each location. The pairwise correlation defined the pattern consistency index at this location and was assigned to all voxels within the respective searchlight sphere region of interest. The mean value was calculated for voxels which were located in overlapping searchlight spheres in order to obtain a representational consistency map.

In order to assess statistical significance of this representational consistency map, it was transformed into percent rank with respect to the pooled distribution of DSM correlations from all the pairwise consistency maps (see Supplementary Figure 1). This thresholding procedure has proven to be robust compared to classical testing in respect to the exponentially growing number of pairwise comparisons and baseline shift of the correlation coefficients. The thresholding procedure was independently conducted for results of the linear and non-linear aligned data. The resulting map was thresholded at a percent rank of 99% corresponding to a mean correlation coefficient of *r*=0.084 for linear alignment and *r*=0.096 for non-linear alignment (for comparison see Table 4 (available online only) and Supplementary Figure 1).

For comparison, a voxel-wise inter-brain correlation was determined for all 153 possible pairs as the Pearson correlation of the time courses for all anatomically corresponding voxels (degrees of freedom=3597). The thresholding is concurrent with the thresholding procedure of the consistency analysis. The thresholds at a percent rank of 95% were *r*=0.077 and *r*=0.086 for linear and non-linear alignment respectively (see Table 5 (available online only)).

**Figure 5:**
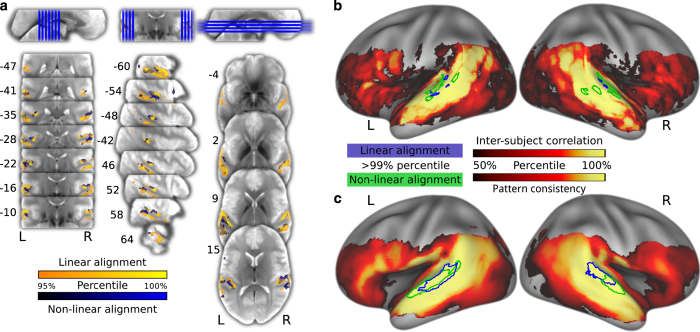
Area of maximum inter-individual response similarity. (**a**) Representative slices of the group EPI template depicting the 95% percentile of univariate inter-brain time-series correlations for linear (blue) and non-linear (yellow) alignment in the group EPI template space. (**b**) Distribution of percent ranks of mean inter-brain correlations on the cortical surface. The statistical map is thresholded at percent rank=50%. The colored outlines depict the 99% percentile for linear (blue) and non-linear (green) alignment. (**c**) Analog to panel B, depicting the distribution of multivariate 2nd-order pattern consistency. Projection onto the surface was performed using Caret 5.64 with the PALS-B12 atlas^40^.

After thresholding, the representational consistency map reveals two clusters in the anterolateral part of the Planum Temporale (PT) in both hemispheres (see Table 6 (available online only) and Figure 5c). The location of the clusters for the univariate inter-individual-correlation confirm the functional contribution of these areas for auditory cognition (see Table 7 (available online only) and Figure 5a/b). The PT, as part of the auditory ventrolateral pathway, is known to be involved in processing complex sounds and speech^38^.

## Usage Notes

The procedures we employed in this study resulted in a dataset that is highly suitable for automated processing. Data files are organized according to the standards implemented by the openfmri.org project^39^. Data are shared in documented standard formats, such as NIfTI or plain text files, to enable further processing in arbitrary analysis environments with no imposed dependencies on proprietary tools. Conversion from the original raw data formats is implemented in publicly accessible scripts; the type and version of employed file format conversion tools are documented. Moreover, all results presented in this section were produced by open-source software on a computational cluster running the (Neuro)Debian operating system^21^. This computational environment is freely available to anyone, and in conjunction with our analysis scripts, it offers a high level of transparency regarding all aspects of the analyses presented herein.

Additional material is available at http://www.studyforrest.org. This includes information on data access options and publications that employ this dataset, as well as source code for data conversion and the processing steps described in this manuscript. The source code also contains a reference implementation for a data access module written in the Python programming language as well as a set of unit tests that verify certain aspects of data integrity and consistency.

All data are made available under the terms of the Public Domain Dedication and License (PDDL; http://opendatacommons.org/licenses/pddl/1.0/). All source code is released under the terms of the MIT license (http://www.opensource.org/licenses/MIT). In short, this means that anybody is free to download and use this dataset for any purpose as well as to produce and re-share derived data artifacts. While not legally required, we hope that all users of the data will acknowledge the original authors by citing this publication and follow good scientific practice as laid out in the ODC Attribution/Share-Alike Community Norms (http://opendatacommons.org/norms/odc-by-sa/).

## Additional information

Tables 4, 5, 6, 7 are only available in the online version of this paper.

**How to cite this article:** Hanke, M. *et al.* A high-resolution 7-Tesla fMRI dataset from complex natural stimulation with an audio movie. *Sci. Data* 1:140003 doi: 10.1038/sdata.2014.3 (2014).

## Supplementary Material



Supplementary Figure 1

## Figures and Tables

**Table 1:** Parts of the original “Forrest Gump” movie that comprise the actual stimulus.

**Part**	**Start time**	**End time**	**Start frame**	**End frame**
0	00:00:00.00	00:21:32.12	0	32312
1	00:24:13.24	00:38:31.23	36349	57798
2	00:38:58.20	00:57:19.22	58470	85997
3	00:59:31.17	01:18:14.00	89293	117351
4	01:20:24.16	01:34:18.06	120616	141457
5	01:37:14.19	01:41:30.19	145869	152269
6	01:42:49.19	02:09:51.17	154244	194792

**Table 2:** Questionnaire items: Demographics, musical background, stimulus exposure.

**Item**	**Description**
id	participant ID
gender	male: m; female: f
age	age group; five years width
handedness	right: r; left: l
german_(speak,comprehend,read,write)	
english_(speak,comprehend,read,write)	language proficiency
french_(speak,comprehend,read,write)	none: 0; notions: 1; can get by: 2; fluent: 3
language4_(speak,comprehend,read,write)	
hearing_problems_past	any hearing problems in the past (middle ear infections, otosclerosis, otospongiosis, hard of hearing, etc.): yes/no
hearing_problems_current	current hearing deficit: yes/no
music_training_harmonics	
music_training_compositionmusic_training_analyticsmusic_training_historymusic_training_other	You have received formal musical training in ear training, harmony, composition analysis, music history, other (e.g., sound engineer, tuner, etc.), improvisation
music_training_improv	How good are you at identifying pitches absolutely?
absolute_pitch	excellent: 3; ok: 2; can't do it: 1
melodic_stability	You can carry a tune: Well: 3; Not very well: 2; Not at all: 1
listen_preference(1–3)_(genre,duration)	Musical genre preference for active listening at home. 1–3 is order of preference. Duration is coded as hours/week.
backg._preference(1–3)_(genre,duration)	Musical genre preference for passive listening while doing other things. 1–3 is order of preference. Duration is coded as hours/week.
concert_preference(1–3)_(genre,duration)	Musical genre preference for concert listening. 1–3 is order of preference. Unit of duration is explicitly specified.
favorite_music_examples	Please give examples of pieces, musicians and composers you listen to the most regularly
musician	You consider yourself to be a musician: yes/no
music_lover	If you are a non-musician, do you consider yourself to be a “music lover”?: yes/no
musician_style(1–3)	If you are a musician: if you sing or play an instrument (or have you sung or played in the past), what style(s) of music did you play? (indicate in order of importance)
musician_instruments	Please indicate all instruments played
musician_improv	Do you improvise: yes/no
musician_improv_style	Improvision style
musician_conductor	Do you conduct?: yes/no
musician_other_activities	Other types of musical activity, please specify.
music._act(1–3)_(,dur.,startage,endage)	You practise or play or have practised or played (answer for each instrument or musical activity). Start/end age is in years. Time involved/duration is coded in hours/week.
forrest_seen	Have you seen the movie “Forrest Gump” before?: yes/no
forrest_seen_dist	How long since you last saw this movie? Time coded in months.
forrest_seen_count	How often have you seen this movie before?
forrest_seen_languages	What languages have you seen this movie in?
forrest_ad_known	Have you heard the audio movie version before?: yes/no
forrest_ad_rating	How did you like the audio movie?:bad: 1; rather bad: 2; rather good: 3; good: 4
forrest_ad_storydepth	How far did you delve into the story?:not at all: 1; a little: 2; rather deep: 3; very deep: 4
forrest_ad_fatigue	Did you get tired during the movie?:not at all: 1; a little: 2; rather tired: 3; very tired: 4
forrest_ad_feeling	How did you feel during the recording session?:bad: 1; rather bad: 2; rather good: 3; good: 4
forrest_ad_artist_count	In your opinion, music from how many artists was played during the movie?
audioq(1--8)	Reported audio quality for each movie segment:very poor: 1, rather poor: 2, rather good: 3, very good: 4

**Table 3:** Overview of known data acquisition anomalies (F: functional data, P: physiological recordings during fMRI session, S: structural data).

**Modality**	**Participant**	**Segment**	**Description**
F	4	8	due to an image reconstruction problem, the last 75 volumes for the last movie segment are missing
F	4	5–8	motion caused by the participant coughing occasionally
F	10	1–8	distortion correction invalid: only non-distortion-corrected fMRI data available
F	11, 13	3, 4, 7, 8	both participants show significant motion due to physical discomfort in the last two segments of both recording sessions (first session in particular); see also Figure 4b
P	1, 2	1–8	recorded at 100 Hz, some intermediate trigger pulses
P	18	1	where not detected and have been interpolated
P	4	1–4	suboptimal data quality – hand with pulse oxymeter sensor was positioned on the participant's stomach and affected by breathing motion
P	7	1–4	pulse oxymeter repeatedly reported bad signal quality
S	2		due to a data IO error 13 slices of the angiography scan are lost (image usable but smaller FoV in Z-direction)
S	6		FoV of the angiography scan had to be enlarged to prevent fold-in effects

**Table 4:** Representational consistency: Spearman *r* at percentiles of the pooled correlation distributions from all the pairwise consistency maps and the mean consistency maps for linear and non-linear anatomical alignment

**Percentile**	**0.0%**	**25.0%**	**50.0%**	**75.0%**	**90.0%**	**95.0%**	**99.0%**	**99.5%**	**100.0%**
*Linear*									
pooled	−0.006	0.004	0.007	0.010	0.021	0.037	0.084	0.106	0.344
mean	0.002	0.005	0.007	0.010	0.020	0.034	0.074	0.089	0.129
									
*Non-linear*									
pooled	−0.006	0.004	0.006	0.009	0.022	0.041	0.097	0.122	0.359
mean	002	0.005	0.006	0.009	0.022	0.040	0.086	0.102	0.153

**Table 5:** Voxel-wise inter-individual correlation: Pearson *r* at percentiles of the pooled voxel-wise inter-individual correlation distributions and the mean correlation maps for linear and non-linear anatomical alignment

**Percentile**	**0.0%**	**25.0%**	**50.0%**	**75.0%**	**90.0%**	**95.0%**	**99.0%**	**99.5%**	**100.0%**
*Linear*									
pooled	−0.469	−0.014	0.008	0.031	0.055	0.076	0.145	0.182	0.694
mean	−0.009	0.002	0.006	0.014	0.030	0.044	0.077	0.090	0.177
									
*Non-linear*									
pooled	−0.412	−0.013	0.008	0.031	0.056	0.078	0.163	0.207	0.719
mean	−0.008	0.002	0.005	0.013	0.032	0.052	0.103	0.126	0.269

**Table 6:** Representational consistency map: Volume of the clusters (Vol) in mm^3^, maximal percent rank (pr), the minimal (Min), median (Med) and maximum (Max) Spearman *r* over the cluster voxels of the mean correlation maps and coordinates of the centre of gravity in the MNI-space for linear and non-linear anatomical alignment; pr>99%. Clusters are displayed in Figure 5c

**Area**	**Vol**	**pr**	***r*** **Min**	**Med**	**Max**	**x**	**y**	**z**
*Linear*								
Planum temporale	3046	99.8	0.084	0.100	0.129	−56.5	−28.3	5.7
Planum temporale	1601	99.5	0.084	0.092	0.110	58.1	−24.5	10.0
								
*Non-linear*								
Planum temporale	3906	99.8	0.097	0.113	0.153	−59.6	−21.4	4.1
Planum temporale	1127	99.5	0.097	0.106	0.125	60.8	−14.2	3.4

**Table 7:** Voxel-wise inter-individual correlation: Volume of the clusters (Vol) in mm^3^, maximal percent rank (pr), the minimal (Min), median (Med) and maximum (Max) Pearson *r* over the cluster voxels of the mean correlation maps, and coordinates of the centre of gravity in the MNI-space for linear and non-linear anatomical alignment; Cluster volume ≥50 mm^3^, pr>95%; Clusters are displayed in Figure 5b

**Area**	**Vol**	**pr**	***r*** **Min**	**Med**	**Max**	**x**	**y**	**z**
*Linear*								
Planum temporale	3046	99.3	0.076	0.093	0.166	−55.1	−32.0	5.9
Planum temporale	2867	99.2	0.076	0.090	0.157	55.7	−29.0	10.6
Angular gyrus	176	97.7	0.076	0.084	0.105	−58.7	−55.6	15.7
Brodmann area 44	153	98.2	0.076	0.084	0.118	−56.0	11.7	11.1
Superior temporal gyrus	69	97.0	0.076	0.084	0.097	−57.2	−21.7	−5.3
Middle temporal gyrus	50	97.6	0.076	0.085	0.107	−52.5	−3.3	−19.5
								
*Non-linear*								
Planum temporale	8826	99.7	0.078	0.109	0.234	−56.1	−27.6	3.0
Planum temporale	6934	99.7	0.079	0.101	0.249	55.5	−25.0	4.4
Angular gyrus	137	96.8	0.079	0.084	0.098	54.6	−52.5	23.4
Angular gyrus	69	97.0	0.080	0.089	0.102	−59.2	−54.9	22.3
Brodmann area 44	61	97.4	0.079	0.086	0.109	−56.9	17.8	6.9
Angular gyrus	59	96.4	0.079	0.083	0.093	−43.5	−59.8	29.1
