# Supplementary material for: A high-resolution 7-Tesla fMRI dataset from complex natural stimulation with an audio movie
Source: Sci Data. 2014 May 27;1:140003. doi: 10.1038/sdata.2014.3 (PMC4322572; doi:10.1038/sdata.2014.3)
Supplement: Supplementary Figure 1 [file sdata20143-s2.pdf]

A high-resolution 7-Tesla fMRI dataset from complex natural stimulation with an audio movie

Supplementary material

Michael Hanke, Florian J. Baumgartner, Pierre Ibe, Falko R. Kaule, Stefan Pollmann, Oliver Speck, Wolf Zinke, Jörg Stadler

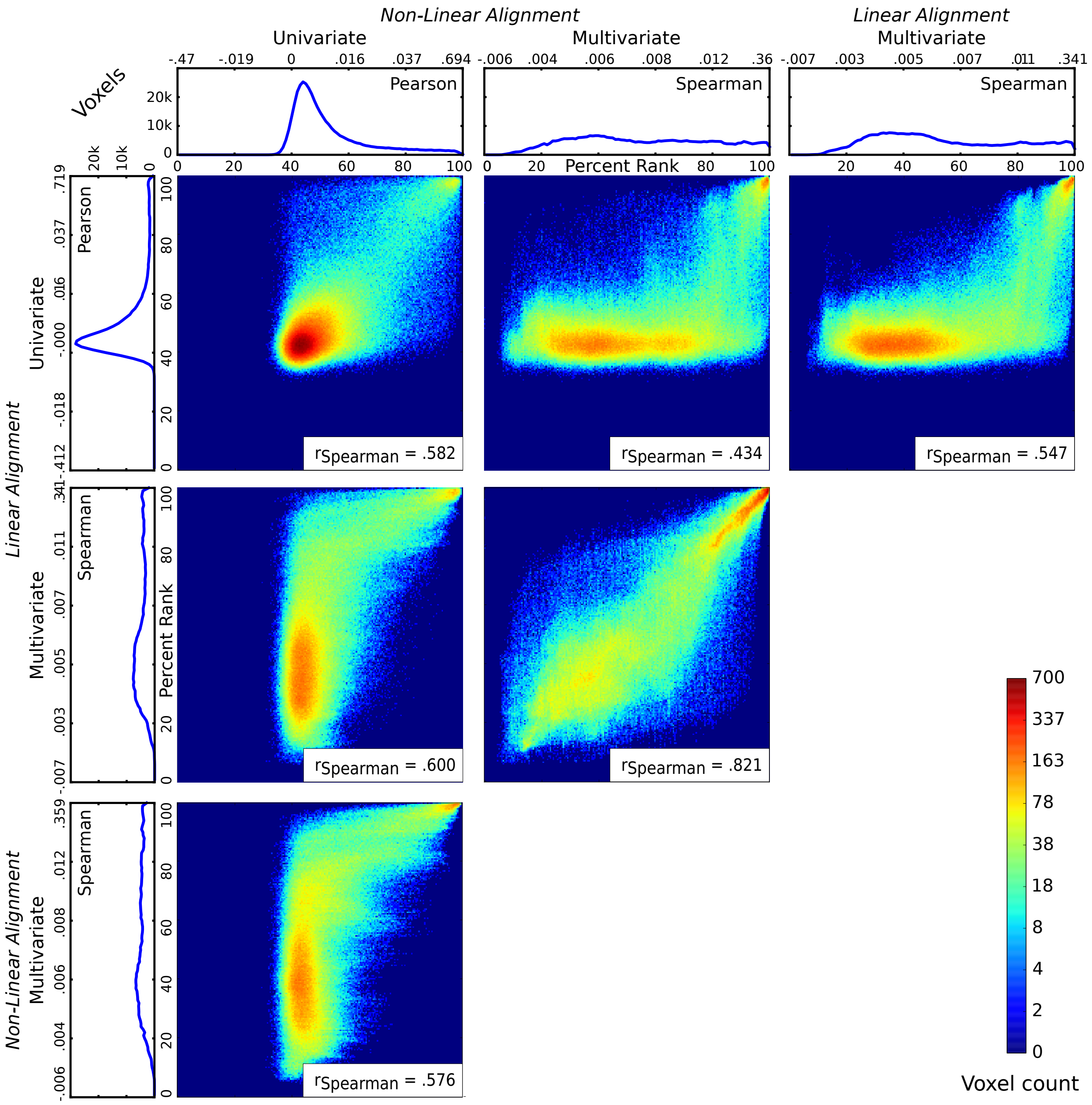

Supplementary Figure 1: Spatial correspondence of the mean multivariate representational consistency map and the univariate inter-individual correlation map for linear and non-linear anatomical alignment. The graphs show the distribution of the percent ranks and the corresponding Pearson and Spearman coefficient for the uni- and multivariate mean map respectively. The 2D histograms illustrate spatial covariance of two distributions. Spearman correlation over voxel specific percent ranks pairs estimates the spatial correspondence.
